# Physical activity, screen time and sleep among children and adolescents: findings from the 2022 active healthy kids Ethiopia

**DOI:** 10.1186/s12887-025-05785-4

**Published:** 2025-07-05

**Authors:** Chalchisa Abdeta, Alem Deksisa, Mesfin Hailu, Debrework Tesfaye, Lucy Westerman, Anthony D. Okely, John J. Reilly, Mark S. Tremblay

**Affiliations:** 1 Early Start, School of Education, University of Wollongong, Wollongong, Australia; 2 Department of Public Health, Adama Hospital Medical College, Adama, Ethiopia; 3 Department of Sport Science, Wolaita Sodo University, Adama, Ethiopia; 4 School of Population and Global Health, University of Melbourne, Melbourne, Australia; 5 Early Start, School of Health and Society, University of Wollongong, Wollongong, Australia; 6 Department of Psychological Sciences and Health, University of Strathclyde, Strathclyde, Scotland; 7 Healthy Active Living and Obesity Research Group, Children’s Hospital of Eastern Ontario Research Institute, Ontario, Canada

**Keywords:** Movement behaviours, Surveillance, Youth, Global matrix, Physical fitness

## Abstract

**Background:**

Few studies have been conducted on movement behaviours in low-income countries, indicating a need for further surveillance. This study aimed to track changes since the release of Ethiopia’s 2018 Report Card indicators and explore further gaps in physical activity, sedentary screen time and sleep among Ethiopian children and adolescents (5–17 years).

**Methods:**

We reviewed studies examining physical activity, sedentary screen time and sleep among Ethiopian children and adolescents. Relevant data were systematically searched from digital databases including PubMed, Medline, Scopus, Web of Science, WHO Hinari and Google Scholar, in alignment with the study objective. Policy or program documents were obtained from Ethiopian government official websites. Records were screened by two independent reviewers and extracted by the first author and verified by a co-author. Data were synthesised according to the harmonised *Active Healthy Kids Global Alliance* standards (A ≥ 80%, B 60–79%, C 40–59%, D 20–39%, F < 20%, INC = incomplete data).

**Results:**

We found eight studies (*n* = 8) with relevant information; all were based on parent- or self-reported data. Only a small proportion of them met the guidelines for physical activity (16%) and sedentary screen time (55%). There were no data available for sleep. Since the release of the 2018 Report Card, there have been improvements in the grades for *School*,* Active Transportation*,* Sedentary Behaviour*,* Community and Environment*, and *Government* indicators from D to A-, C to B-, F to C+, F to C-, and D to C, respectively. However, grades for *Overall Physical Activity*, and *Organised Sport and Physical Activity* decreased from D to F and from C to C-, respectively, while the rest of the indicators remained unchanged. The *Sleep* indicator was introduced for the first time.

**Conclusion:**

Some indicators highlighted positive changes but limitations with data representativeness and quality underscore the need for improved surveillance to understand and promote healthy levels of movement behaviours in Ethiopian children and adolescents.

## Background

Adequate physical activity and sleep, along with reduced sedentary behaviour including screen time are beneficial for health and well-being in children and adolescents [1–5]. The World Health Organization (WHO) recommends that children and adolescents aged 5–17 years should attain: (i) At least an average of 60 min of moderate-to-vigorous intensity physical activity per day; (ii) Vigorous-intensity physical activity and muscle and bone strengthening activities at least 3 days per week; and (iii) Limit sedentary time, particularly sedentary recreational screen time [6]. However, the WHO’s 2020 guidelines lack specific recommendations for screen time and sleep [6]. Comparable Canadian guidelines advise that children and adolescents aged 5–17 years should spend less than two hours a day on recreational screen time. They also encourage limiting time spent in extended sitting and replacing sitting time with physical activity. Moreover, the Canadian guidelines suggest that children aged 5–13 years should get 9–11 h of good quality sleep per 24-hour period, while adolescents aged 14–17 years should have 8–10 h of good quality sleep during the night [3].

Over 80% of children and adolescents worldwide (ages 11–17 years) fail to meet the WHO recommendations for physical activity, putting them at higher risk for non-communicable diseases (NCDs) [7, 8]. Physical inactivity, sedentary behaviour, and inadequate sleep are growing concerns for NCDs globally [9], now the leading causes of disability and premature death, affecting families, communities, health systems, and economies [10]. Additionally, physical inactivity is a major risk factor for childhood obesity, which is on the rise globally [11]. Promoting healthy levels of these behaviours can significantly enhance health outcomes, contributing to the *United Nations Sustainable Development Goal (SDG) 3: Good Health and Well-being* by reducing NCDs and improving overall health [12]. Increased physical activity also helps to improve cognitive function, concentration, and memory in children and adolescents, leading to better academic performance and overall educational attainment [6], aligning with *SDG 4: Quality Education*. Furthermore, addressing these issues can help reduce health disparities and promote social equity, supporting *SDG 10: Reduced Inequalities*. Additionally, initiatives like promoting active transport and community sports can contribute to *SDG 11: Sustainable Cities and Communities* by creating more sustainable and equitable urban environments. These efforts collectively aim to reduce NCDs, enhance education, reduce inequalities, and create healthier communities, demonstrating the interconnected nature of the SDGs [12–14].

There’s a paucity of evidence in global surveillance for movement behaviours and their determinants especially in Low-and Middle-Income Countries (LMICs) [13]. The *Active Healthy Kids Global Alliance (AHKGA)* is intensifying efforts globally to address this disturbing evidence gap through a surveillance initiative called the ‘*Global Matrix’* that enables engagement of LMICs in surveillance [9, 15]. The AHKGA enables countries to monitor and compare children and adolescent’s physical activity, sedentary behaviour, and sleep through harmonised development of country Report Cards (RC) [9]. In 2018, Ethiopia released the first country RC [16]. Tracking changes since this inaugural RC can provide valuable insights on physical activity, sedentary screen time and sleep among children and adolescents. Full reports on Ethiopia’s participation can be found here. This information can inform policy and practice, aiding the implementation of the WHO Global Action Plan on Physical Activity 2018–2030 in Ethiopia and other LMICs [8, 17]. This study aimed to track changes since the release of Ethiopia’s 2018 RC indicators and explore further gaps in physical activity, sedentary screen time and sleep among Ethiopian children and adolescents (5–17 years).

## Methods

### Study design, setting and participants

We reported following the updated Preferred Reporting Items for Systematic reviews and Meta-Analyses (PRISMA) guidelines [18]. This study was conducted in Ethiopia, the second most populous nation in Africa with a population exceeding 120 million people. It is notable that about 80% of this population resides in rural areas, reflecting the country’s predominantly agrarian lifestyle. Ethiopia is categorised as a low-income country, and economically relies on agriculture [19]. However, rapid rural-urban migration is anticipated to increase the urban population from 21% in 2018 to 39% by 2050 [20]. This shift might induce various changes in physical activity, sedentary behaviour and sleep due to limited equitable infrastructure such as footpaths, youth centres, and recreational spaces [21]. Additionally, increased access to electronic devices might encourage children and adolescents to spend more time on screens in urban areas [22]. Recognising the importance of movement behaviour surveillance among children and adolescents, the country RC leader (CA) took the initiative to register Ethiopia to participate in the Global Matrix 4.0 study. Ethiopia’s RC team formed (AD, MH, and DT) with multidisciplinary expertise in public health, physiotherapy, physical activity, and sport science. This study drawn from studies included children and adolescents aged 5–17 years old in Ethiopia who were apparently healthy and had no known health problems or disabilities. Data were collected between January 1, 2018, and August 30, 2022.

### Eligibility criteria

The search terms were built on the *Population*,* Concept and Context (PCC) framework* [22]. Studies were included if they reported data on physical activity, sedentary screen time and sleep among Ethiopian children and adolescent (5–17 years old or any subset), either separately or together. Studies published between January 2018 and August 2022 were included. Studies focused on children and adolescents with known health problems or disabilities as well as participants over 17 years were excluded.

### Data sources

Table 1 presents data sources used in this study. This study was guided by systematically searched data from digital databases including PubMed, Medline, Scopus, Web of Science, WHO Hinari and Google Scholar, in alignment with the study objective. Policy or program documents were obtained from Ethiopian government official websites, including the Ministry of Health, Ministry of Women, Children and Youth, and Ministry of Culture and Sport. The AHKGA benchmarks were used to assign grades for the RC indicators.

**Table 1:** Data sources for Ethiopia’s 2022 report card development

Indicator	Average taken	Study characteristics						
		Individual findings	Study design	Sample (*n*)	Age (years)	Nature of study	How data measured	Reference
Overall Physical Activity	16%	14%	Cross-sectional	*n* = 632	5–18	Parent- or self-reported	Proportion of participants who met at least 60 min of moderate or vigorous aerobic activity daily, including vigorous activity and muscle-strengthening exercises at least 3 days a week).	Biadgilign et al., 2022
		17%	Cross-sectional	*n* = 580	13–19		Proportion of participants who met 60 min of moderate to vigorous physical activity per day for at least three days per week during recreation, sport, and leisure-time.	Mohammed et al., 2020a
Organized Sport and Physical Activity	44%	63%	Cross-sectional	*n* = 498	10–19		Proportion of participants engaged in moderate-to-vigorous sports activities per week.	Belay et al., 2021
		26%	Cross-sectional	*n* = 616	6–12		Proportion of participants involved in Sport activity per week.	Mekonnen et al., 2018
Active Play	68%	77%	Cross-sectional	*n* = 482	10–18		Proportion of children and adolescents who regularly played in their home compound.	Fitsum et al., 2021
		58%	Cross-sectional	*n* = 580	13–19		Proportion of children and adolescents who had access to playground.	Mohammed et al., 2020a
Active Transportation	68%	32%	Cross-sectional	*n* = 498	10–19		Proportion of participants who walked or biked to and from school on weekdays.	Belay et al., 2021
		76%	Cross-sectional	*n* = 482	10–18			Fitsum et al., 2021
		80%	Cross-sectional	*n* = 522	10–19			Worku et al., 2021
		82%	Cross-sectional	*n* = 616	6–12			Mekonnen et al., 2018
Sedentary behaviour	54%	44%	Cross-sectional	*n* = 482	10–18		Proportion of participants who spent time on watching movies/TV for more than 2 h per day.	Fitsum et al., 2021
		65%	Cross-sectional	*n* = 580	13–19		Proportion of participants who spent time on screen devices for more than 2 h per day.	Mohammed et al., 2020b
Physical Fitness	-	-	-	-	-	-	-	No clear evidence
Sleep	-	-	-	-	-	-	-	No clear evidence
Family and Peers	*AHKGA benchmark (< 20%)	14%	Cross-sectional	-	5–17	Parent- or self-reported	Proportion of children and adolescents who receive encouragement and support from their family and friends to engage in physical activity.	Abdeta et al., 2019
School	*AHKGA benchmark (80-86%)	82%	Cross-sectional	*n* = 632	5–18		Proportion of children and adolescents who participated in physical education at school.	Biadgilign et al., 2022
Community and Environment	43%	58%	Cross-sectional	*n* = 580	13–19		Proportion of participants with access to a playground in their residential area.	Mohammed et al., 2020b
		28%	Cross-sectional	*n* = 580	13–19		Proportion of participants with access to a gymnasium in their residential area.	Mohammed et al., 2020b
Government	*AHKGA benchmark (47 − 53%)	Car Free Day initiative	-	-	-	Monthly event	Presence of documented evidence of policies and strategies addressing physical activity, along with their implementation.	Government report
		Mass Sports campaign	-	-	-			Government report; Tulu et al., 2019
		Non-Motorised Transport Strategy 2020–2029	-	-	-	10 years		Government report
		National Adolescent and Youth Health Strategy (2016–2020)	-	-	-	5 years		

*AHKGA: Active Healthy Kids Global Alliance. The percentage column represents the percentage of children and adolescents who meet the AHKGA benchmark. All data were derived from individual studies published between January 2019 and August 2022, following the last report card

Table 2 displays the standard grading system for the AHKGA indicators. We used the ten core harmonised indicators for physical activity (*Overall Physical Activity*, *Organised Sport and Physical Activity*, *Active Play*, *Active Transportation*, *Physical Fitness*, *Family and Peers*, *Schools*, *Community and Environment*, and *Government*) and *Sedentary Behavi*our. Additionally, the *Sleep* indicator was added for the first time to expand Ethiopia’s 2018 RC. The Canadian 24-hour movement guidelines for children and youth were used for the sleep indicator [3].

**Table 2:** Report card grading rubric.*

Grade	Benchmarks	Explanation
A	A^+^=94 − 100% A = 87 − 93% A^−^ = 80 − 86%	We are succeeding with a large majority of children and adolescents (≥ 80%)
B	B^+^=74 − 79% B = 67 − 73% B^−^ = 60 − 66%	We are succeeding with well over half of children and adolescents (60 − 79%)
C	C^+^=54 − 59% C = 47 − 53% C^−^ = 40 − 46%	We are succeeding with about half of children and adolescents (40 − 59%)
D	D^+^=34 − 39% D = 27 − 33% D^−^ = 20 − 26%	We are succeeding with less than half with some children and adolescents (20 − 39%)
F	F = 0 -19%	Wearesucceeding with veryfew of children and adolescents (< 20%)
INC	INC = Incomplete	Inadequate information to assign a grade

*Taken from Active Healthy Kids Global Alliance

### Data analysis

Data were analysed using harmonised procedures of the AHKGA guidelines and indicators [9]. The country RC team evaluated the existing available evidence and assigned grades for all indicators and sent it to AHKGA auditors for approval. The average individual prevalence findings for each indicator were computed to assign grades based on the AHKGA benchmarks. Grades were determined by the percentage of Ethiopian children and adolescents who met the benchmark for each indicator. Each grade reflected the country’s success in creating opportunities that encourage children and adolescents to engage in physical activity and sleep well, while reducing sedentary screen time behaviour. Progress was evaluated by comparing the 2022 grades with those from the 2018 RC to identify areas where further improvement is required.

## Results

### Source of evidence and report card grades

**Fig. 1:**
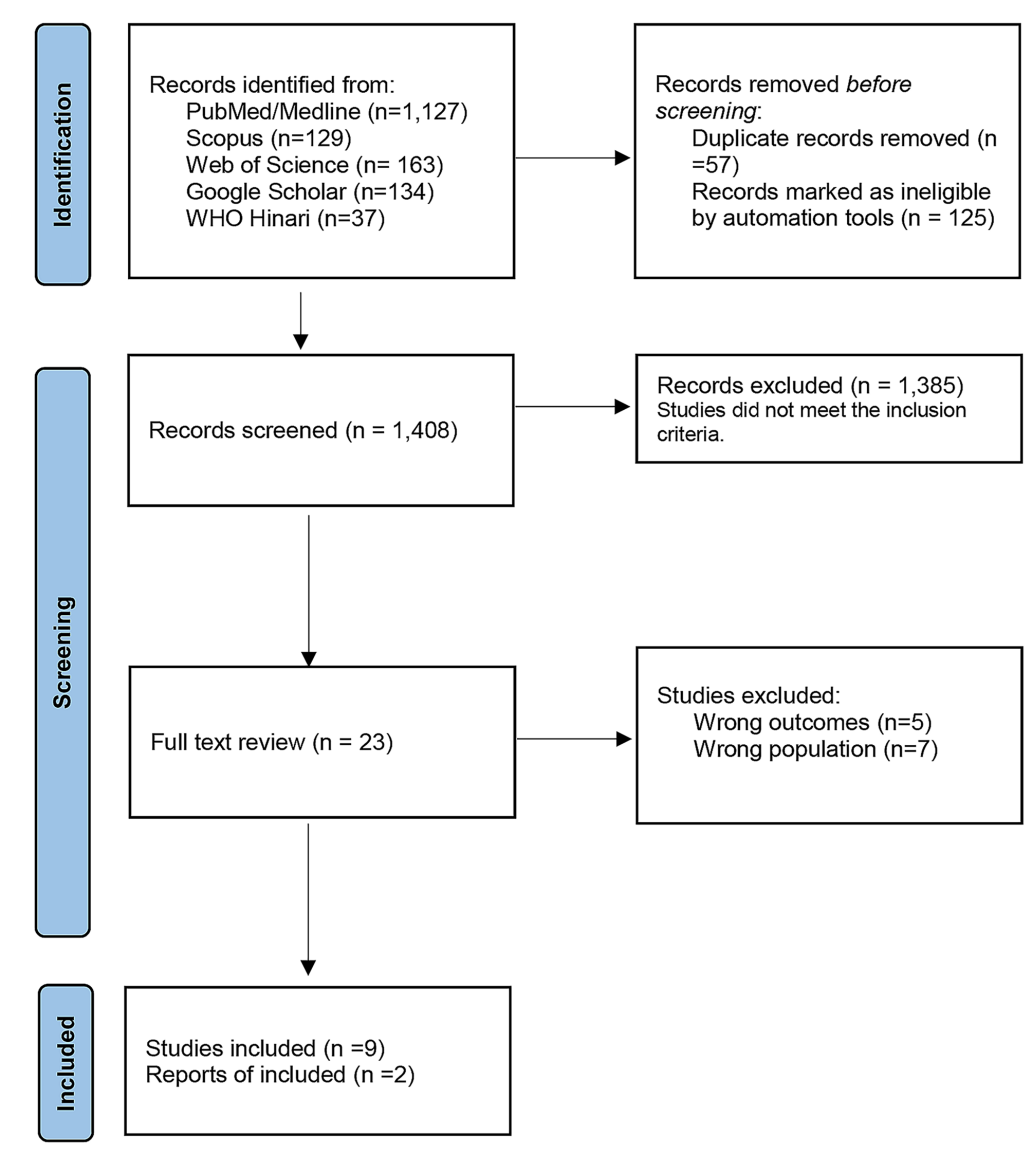
PRISMA flowchart diagram

Figure 1 illustrates the PRISMA flowchart for this review, while Table 3 presents the RC grades. Data used for grading all indicators were primarily derived from individual studies due to a dearth of evidence, and all of them were based on parent-or self-reports. We only found nine studies and two government documents related to the physical activity of children and adolescents in Ethiopia.

**Table 3:** Summary of Ethiopia’s 2022 report card

Indicator	Grade	Metrics used to assign a grade	Rationale for Grade	2018 grades	Recommendations to improve the grades
Overall Physical Activity	F	Proportion of children and adolescents who meet the WHO physical activity guideline.	Only 15.8% of Ethiopian children and adolescents met the WHO physical activity guideline (Mohammed et al., 2020a; Biadgilign et al., 2022).	D	• Integrate physical activity questions into the Ethiopia Health and Demographic Survey. • Promote and create more opportunities for physical activity. • Develop locally appropriate physical activity programs.
Organised Sport and Physical Activity	C-	Proportion of Ethiopian children and adolescents participating in organised sport and physical activity at least once per week.	44.1% of Ethiopian children and adolescents were participating in organised sport activities in school environments (Mekonnen et al., 2018; Belay et al., 2021).	C	• Increase organised sports participation within residential and school environments. • Integrate organised sports into the school curriculum.
Active Play	B	Proportion of Ethiopian children and adolescents who engaged in unstructured active play at any intensity for more than two hours a day.	67.7% of Ethiopian children and adolescents were involved in outdoor active play at any intensity for more than 2 h per day (Mohammed et al., 2020a; Fitsum et al., 2021).	B	• Create unstructured outdoor play opportunities in residential areas. • Build more playgrounds in urban and rural areas.
Active Transportation	B-	Proportion of Ethiopian children and adolescents who use active transportation to get to and from places in the form of walking and biking to go to school or friend’s home, or recreational places.	64.0% of Ethiopian children and adolescents were using active forms of transportation (walking/biking) to and from school (Mekonnen et al., 2018; Belay et al., 2021; Fitsum et al., 2021; Worku et al., 2021).	C	• Promote active transportation to create public awareness. • Improve pedestrian and cycling infrastructure. • Develop safe routes for walking and biking to school. • Implement *Ethiopia Non-Motorised Transport Strategy 2020–2029*.
Sedentary behaviour	C+	Proportion of Ethiopian children and adolescents who engage in 2 h or less of sedentary recreational screen- time per day.	On average, the proportion of Ethiopian children and adolescents who met the sedentary recreational screen time guidelines was 54.5% for this indicator (Mohammed et al., 2020b; Fitsum et al., 2021).	F	• Educate families on setting screen time limits. • Promote alternative activities to screen time, such as reading or outdoor play. • Integrate screen time education into the school curriculum to help students make informed decisions.
Physical Fitness	INC	Proportion of Ethiopian children and adolescents who meet criterion-referenced standards for cardiorespiratory fitness, muscular strength, and endurance.	There is no adequate information in Ethiopia to assign a grade for this indicator.	INC	Conduct research on physical fitness levels among children and adolescents.
Sleep	INC	Proportion of Ethiopian children and adolescents who meet the Canadian sleep guidelines.	There is no adequate information in Ethiopia to assign a grade for this indicator.	-	Conduct research on sleep duration and quality among children and adolescents.
Family and Peers	F	Proportion of Ethiopian children and adolescents who get support from their friends, peers, and families to get physically active.	Only 14% of children were receiving encouragement and support from their families and friends to move (Abdeta et al., 2019).	F	• Encourage parents to participate in physical activities with their children and adolescents. • Develop locally appropriate family-based physical activity programs.
School	A-	Proportion of schools with policies and infrastructures that support physical activity participation of Ethiopia’s children and adolescents with trained physical education specialists in the school.	81.5% of children and adolescents in Ethiopia were participating in physical education at school (Biadgilign et al., 2022).	D	• Expand extracurricular physical activity opportunities. • Develop school-based physical activity programs.
Community and Environment	C-	Percentage of communities/municipalities that create opportunities for physical activity among children and adolescents in Ethiopia.	43% of children and adolescents had access to physical activity infrastructure such as playgrounds and gymnasiums (Mohammed et al., 2020a).	F	• Expand public spaces that supports physical activity, such as parks and recreational areas. • Develop community-based physical activity programs.
Government	C	Evidence of government policy and strategies that allocate resources to support and implement physical activity initiatives for children and adolescents in Ethiopia.	Considering changes made on physical activity strategies and campaigns in Ethiopia, the Report Card team assign grade ‘C’ for this indicator (MOH, 2016;2019; Tulu et al., 2019; AACA, 2020).	D	• Develop comprehensive national movement behaviours guidelines • Improve movement behaviours surveillance through regular national surveys. • Implement the WHO Global Action Plan for Physical Activity 2018–2030 in Ethiopia (WHO, 2018). • Expanding existing national initiatives such as *Car Free Day* and *Mass Sport program*

Since the 2018 RC, there have been slight improvements in some grades, but not others. The grades for the *Overall Physical Activity* and *Organised Sport and Physical activity* indicators decreased from D to F and from C to C-, respectively. The grades for the *Active Play*, *Physical Fitness*, and *Family and Peers* indicators remained unchanged. The *School*,* Active Transportation*,* Sedentary Behaviour*,* Community and Environment*, and *Government* indicators showed an increase in grades from D to A-, C to B-, F to C+, F to C-, and D to C, respectively. The *Sleep* indicator was introduced for the first time.

### Physical activity, sedentary screen time and sleep profiles

Despite some improvements since the 2018 RC, significant limitations and surveillance gaps persist, particularly due to the lack of national data on movement behaviours. We found that physical activity profile of Ethiopian children and adolescents is alarmingly low. However, some positive aspects are worth highlighting. The *School* indicator received an A- for its role, with 82% of children and adolescents participating in physical education. *Active Play* also showed promising results, earning a B grade, as 68% of children engage in unstructured active play for more than two hours daily. *Active Transportation* was graded B-, with 68% of children walking or biking to school. On the other hand, *Organised Sport and Physical Activity*, as well as *Community and Environment* indicators, were graded C-, with just over 40% of children having access to physical activity infrastructure, such as parks or playgrounds, and actively participating in these activities. However, the *Overall Physical Activity*, and *Family and Peers* indicators were notably low, graded F, with only 14–16% of children receiving encouragement and meeting the WHO physical activity guidelines. Unfortunately, the *Physical Fitness* indicator remained ungraded due to insufficient data. Lastly, the *Government* indicator was graded C, indicating that while there are existing policies and strategies, there is a pressing need for improved surveillance, strategies, policies and investment to better support physical activity initiatives. The present study also reported the sedentary screen time profile of Ethiopian children and adolescents. The *Sedentary Behaviour* indicator received a grade of C+. On average, 54% of children and adolescents in Ethiopia met the Canadian sedentary screen time guidelines. Lastly, the *Sleep* indicator was graded incomplete (INC) due to insufficient data.

## Discussion

### Summary of the findings

The present study showed that only a small proportion of Ethiopian children and adolescents met the guidelines for physical activity (16%) and sedentary screen time (55%). Unfortunately, there were no available data for sleep. Since the release of Ethiopia’s 2018 RC, there have been improvements in the grades for *School*,* Active Transportation*,* Sedentary Behaviour*,* Community and Environment*, and *Government* indicators. However, grades for *Overall Physical Activity*, and *Organised Sport and Physical activity* decreased, while the rest of indicators remained unchanged. Despite some improvements since the 2018 RC, significant limitations and surveillance gaps persist, particularly due to the lack of national data on these behaviours.

### Physical activity, sedentary screen time and sleep profiles

This study highlighted the movement behaviour profiles of Ethiopian children and adolescents, noting some progress since the 2018 Report Card, along with persistent challenges. Similar AHKGA indicators and metrics were applied in both report cards, but new evidence was included in the recent RC. An increased grade in *School* (from D to A-), *Active Transportation* (from C to B-), *Community and Environment* (from F to C-), and *Government* (from D to C) indicators are encouraging. These improvements might be attributed to physical education sessions at school [23], limited vehicle ownership that encourages children and adolescents to use active travel (walking or biking) to and from school [24–27], and access to some physical activity infrastructure such as playgrounds [28]. Recent government initiatives that support physical activity in Ethiopia, such as *Car Free Day initiatives*, the *Mass Sport Program*, and the *Ethiopia Non-Motorised Strategy 2020–2029* [29–31], may also have helped, despite some disruptions due to the COVID-19 pandemic and conflicts in the country. However, the decline in grades for *Overall Physical Activity* (from D to F) and *Organised Sport and Physical Activity* (from C to C-) are concerning, as levels of physical activity were so low [23–25, 28], indicating a need for interventions to promote healthy behaviours. Furthermore, grades for the *Active Play* (B), *Physical Fitness* (Incomplete), and *Family and Peers* (F) indicators remained unchanged since the 2018 RC [16]. The *Sedentary Behaviour* indicator improved from F to C+, with 55% of children and adolescents meeting the sedentary screen time guidelines [26, 32, 33]. This suggests some progress in reducing screen time, but more effort is needed to further minimise screen time and promote active alternatives. In the context of a low-income country like Ethiopia, several socio-economic factors contribute to sedentary screen time [34]. Children and adolescents tend to engage in sedentary screen time due to limited access to alternative recreational activities [35]. The affordability and growing availability of electronic devices have made screen time a convenient option for many households in LMICs [36]. The introduction of the *Sleep* indicator was a significant step forward, despite the current lack of information to assign it a grade. One study [23] attempted to report sleep as a correlate of physical activity but did not adequately specify the compliance according to the existing sleep guidelines for this age group [3, 37].

When interpreting our findings, it’s important to consider several factors. Variations in data sources are expected due to updates in the 2020 WHO guidelines [6]. Studies conducted before 2020 were reported based on the 2010 WHO guidelines, which had different recommendations [38]. For example, unlike the 2010 WHO guidelines, the 2020 guidelines recommend an average rather than a fixed threshold, as detailed in the background section. Additionally, some data sources measured physical activity below the standard of either the former or 2020 WHO guidelines. These variations specifically affect the *Overall Physical Activity* indicator. Moreover, it is unclear whether the data sources included educational screen time or only recreational screen time for the *Sedentary Behaviour* indicator. Furthermore, new evidence reported since the release of the 2018 RC might also affect grade changes for all indicators. Such factors could impact grading accuracy, even if we used the average of those data sources to assign a grade. These changes could result from measurement differences rather than actual behavioural changes. However, it remains unclear whether the changes in grades genuinely reflect a true change in behaviours.

### Recommendations

The present study identified critical gaps in physical activity, sedentary screen time, and sleep among Ethiopian children and adolescents. To address these, it is essential to implement targeted interventions and develop national movement behaviour guidelines that promote physical activity and reduce sedentary behaviour, which align with *SDG 3: Good Health and Well-being*, and *SDG 4: Quality Education* by integrating physical activity into school curricula. Further studies are needed, particularly by integrating surveillance of these behaviours into existing national surveys. This supports SDG 3 by monitoring health risks and *SDG 17: Partnerships for the Goals* through collaboration with various stakeholders. Expanding existing government initiatives like *Car Free Day*, *Mass Sport Program*, and the *Ethiopia Non-Motorised Strategy 2020–2029* promotes *SDG 11: Sustainable Cities and Communities* by encouraging non-motorised transport and *SDG 13: Climate Action* by reducing greenhouse gas emissions. Community engagement, and family support are crucial in encouraging healthy habits, contributing to SDG 3 by creating a supportive environment and *SDG 10: Reduced Inequalities* by ensuring all children have access to opportunities for physical activity and healthy living.

### Strengths and limitations

This study provided a valuable snapshot of physical activity and sedentary screen time among Ethiopian children and adolescents. It offered detailed data, identified critical gaps, and included actionable recommendations to inform policy, practice and future surveillance. However, the study has limitations, including insufficient data to accurately grade *Physical Fitness* and *Sleep* indicators, reliance on potentially biased parent- or self-reported data, and the absence of nationally representative data on sleep and other behaviours, preventing a more complete understanding of these behaviours. Additionally, sex or gender differences were not examined, which impacts the assessment of health equity. Furthermore, the current study is limited to available data sources, and future research should consider utilising primary data sources to provide a more accurate and complete understanding.

## Conclusion

The best available data reveals significant room for improvement in physical activity, sedentary screen time, and sleep among Ethiopian children and adolescents. Addressing these issues will require policy, surveillance, and program actions that promote healthy levels of movement behaviours in Ethiopia.

## Data Availability

The datasets used and/or analysed during the current study are available from the corresponding author on reasonable request.

## Abbreviations

AHKGA: Active Healthy Kids Global Alliance
LMICs: Low- and Middle-Income Countries
NCDs: Non-Communicable Diseases
PCC: Population, Concept, and Context
RC: Report Card
SDGs: Sustainable Development Goals
STROBE: Strengthening the Reporting of Observational Studies in Epidemiology
WHO: World Health Organization
